# Comprehensive Characterization of Bioactive and Undesirable Compounds in Mezcal-Derived Vinasse for Potential Circular Applications

**DOI:** 10.3390/foods15091569

**Published:** 2026-05-02

**Authors:** Alejandro Castrejon, Jimena Álvarez-Chávez, Marcela Gaytán Martínez, Elisa Dufoo-Hurtado, Juan Luis de la Fuente, Héctor Emmanuel Cortés-Ferré, Mar Villamiel, Aurea K. Ramírez-Jiménez

**Affiliations:** 1 School of Engineering and Sciences, Tecnologico de Monterrey, Avenida Eugenio Garza Sada 2501 Sur, Monterrey 64849, NL, Mexico; 2 Posgrado en Ciencia y Tecnología de los Alimentos, Research and Graduate Studies in Food Science, School of Chemistry, Universidad Autónoma de Querétaro, Centro Universitario, Santiago de Querétaro Qro 76010, CP, Mexico; 3 Grupo de Química y Funcionalidad de Carbohidratos y Derivados, Instituto de Investigación en Ciencias de la Alimentación, CIAL (CSIC-UAM), 28006 Madrid, Spain

**Keywords:** agave waste, vinasse, phenolic compounds, furfural, antioxidant activity, circular economy

## Abstract

The mezcal industry in Mexico generates substantial volumes of vinasse, a waste product rich in organic material and bioactive compounds, yet its environmental impact and potential valorization in the food and biotechnological field remain underexplored. This study presents a comprehensive physicochemical and functional characterization of mezcal vinasse derived from mezcal production, including antioxidant activity and cytotoxicity assessment. Proximate analysis revealed high moisture content (96%) and a carbohydrate-rich profile (87.58% dry basis), with notable fiber fractions predominantly composed of insoluble dietary fiber (9.10% dry basis). Low-molecular-weight carbohydrate analysis identified fructose (60.46%) and glucose (10.48%) as the major components, and the hydrolyzed sample showed a monomeric profile with arabinose (31.98%) and glucose (24.14%) as the predominant sugars. Vinasse was found to provide antioxidant activity, as assessed by DPPH (296.3 µmol TE/g) and ABTS (465.3 µmol TE/g) colorimetric assays. Undesirable and antinutritional compounds such as tannins (15.3 mg catechin/g), oxalates (14.6 mg sodium oxalate/g), hydroxymethyl furfural (HMF) (3830.0 mg/L), and furfural (160.0 mg/L) were also quantified, highlighting potential environmental and nutritional concerns due to its mutagenic character at high concentrations. Despite these challenges, vinasse exhibited no cytotoxicity in Caco-2 cells at tested concentrations (25 to 100 mg/mL of phenolic extract), suggesting feasibility for further biotechnological applications.

## 1. Introduction

The word mezcal is used to refer to the distilled beverage produced by fermenting various species of agave in one of the 26 states of Mexico, covered by the Protected Designation of Origin (PDO) for this product [1]. Unlike tequila, which is exclusively produced from *Agave tequilana* Weber var. azul under standardized production conditions, mezcal production utilizes a variety of agave species, such as *A. angustafolia*, *A. durangensis* and *A. salmiana*, as well as wild agaves collectively referred to as “criollo agave” [2,3]. These differences are expected to result in distinct physicochemical compositions. Therefore, studies based on tequila-derived systems may not accurately represent their mezcal analogs.

**Figure 1:**
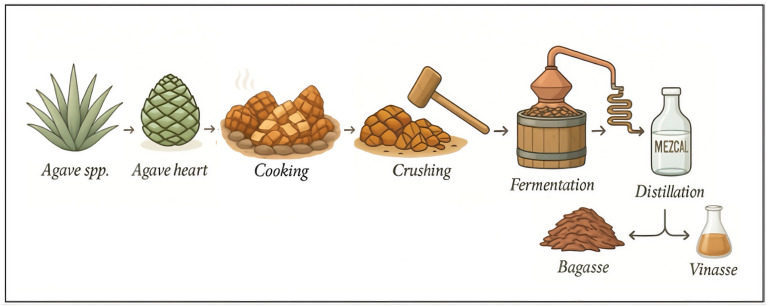
Mezcal process and vinasse generation. Image partially generated using ChatGPT 5.0.

In recent years, mezcal has gained significant attention in the global spirits market. In 2020, mezcal exports reached approximately 4 million liters, and between 2012 and 2020 the total export value of mezcal increased by 603%, reflecting a rapid expansion in international demand [4]. This sustained growth indicates that mezcal has transitioned from a primarily regional product to a globally valued spirit. However, this growth has also brought attention to the environmental challenges posed by this industry, particularly the management of vinasse, a liquid waste of mezcal production (Figure 1). The mezcal industry in Mexico produces approximately 7.5 million liters per year [5]. For every liter of mezcal produced, an estimated 15 L of vinasse are generated, resulting in significant quantities of this waste product [6].

Vinasse is a brown liquid containing organic and bioactive compounds, characterized by its acidity, recalcitrance, and high chemical and biological oxygen demand (COD and BOD) [7,8].

Improper disposal of vinasse, often into water bodies such as rivers and oceans, poses significant environmental concerns [7]. Although legislation, such as NOM-003-SEMARNAT-1997, establishes maximum permissible limits for pollutants in wastewater released to public services, the regulatory framework is insufficiently enforced. This lack of effective waste management practices exacerbates the problem, contributing to the contamination of water sources and highlighting the urgent need for sustainable solutions. Addressing the proper management and utilization of mezcal vinasse is essential to ensure the long-term environmental sustainability of the industry.

Interestingly, recent studies on vinasse have primarily focused on its environmental impact and its potential as a biofuel source, particularly for bio-hydrogen production [9,10,11,12,13]. Nevertheless, research exploring its potential as a prebiotic or food ingredient remains limited, despite the bioactive properties of mezcal vinasse and its rich content of carbohydrates. A critical knowledge gap remains regarding its chemical, nutritional, and functional characterization, particularly in relation to its potential as a consumable resource, whether as an additive, functional ingredient, or other food-related application. This gap may be attributed to the challenges posed by the vinasse matrix, including its low pH, recalcitrant nature, its high COD and BOD, the presence of compounds like hydroxy methyl furfural (HMF), and undesired process-generated components such as oxalates, and tannins [14].

Moreover, most studies on agave by-products have primarily focused on tequila production, leaving mezcal vinasse comparatively underexplored. The differences in production processes and agave species used for mezcal and tequila are expected to generate distinct vinasse compositions, reinforcing the need to study each stream individually.

Additionally, the mezcal used in this study originates from the central part of Mexico, a region not traditionally recognized for mezcal production, further highlighting the novelty and relevance of this research. In this context, the present study provides a comprehensive physicochemical, nutritional, and functional characterization of mezcal vinasse, thereby generating foundational information needed to support its valorization and future applications beyond current practices.

## 2. Materials and Methods

### 2.1. Sample Collection

The mezcal used for vinasse production was obtained from *Agave angustifolia*. Mezcal-vinasse samples were obtained in February 2022 from the mezcal distillery “La Cascada,” located in Malinalco, State of Mexico, at an approximate elevation of 1750 m above sea level. Approximately 5 L of vinasse were collected immediately after the production processes, placed in plastic containers, and subsequently stored at −80 °C in a deep freezer for periods ranging from 1 week to 1 month, depending on the analysis. Prior to experimentation, samples were thawed at 4 °C under standard laboratory conditions.

### 2.2. Proximate Composition

The proximate composition was analyzed using the AOAC [15] methods. Each analysis was performed with three replicates. Method 925.10 was used to determine moisture content; Method 920.39 was employed to quantify lipids; and Method 942.05 was utilized for ash quantification. Method 984.13 was followed for protein quantification, using a nitrogen factor of 6.25. The total carbohydrate content was calculated by difference.

Total fiber, soluble fiber, and insoluble fiber content of the mezcal-vinasse sample were determined using the Fiber Kit from Megazyme “K-TDFR-200A” (Megazyme International, Bray, Wicklow, Ireland), following the manufacturer’s instructions according to AOAC- and AACC-approved methods. Briefly, the sample undergoes enzymatic digestion to remove starch and protein, followed by the separation and quantification of soluble and insoluble fibers. The sample was first treated with heat-stable α-amylase to hydrolyze starch, followed by protease to degrade proteins, and finally amyloglucosidase to break down any remaining polysaccharides. The residue was then filtered and dried to determine the insoluble fiber content. For soluble fiber, the filtrate was precipitated with ethanol, filtered, and dried. The total fiber was calculated by summing the soluble and insoluble fiber fractions. Each analysis was performed in triplicate.

### 2.3. Reducing Sugars

Reducing sugars were quantified using an established colorimetric method. Samples were extracted in aqueous solution with distilled water at a dilution ratio 1:10 (1 g of sample in 10 mL of water). The samples were centrifuged at 10,000 rpm for 30 s to obtain a clear supernatant. Measurements were conducted in triplicate, and the results were expressed as milligrams of sugar per gram of dry weight.

According to Miller [16], reducing sugars were measured using the 2,5-dinitrosalicylic acid (DNS) method. Absorbance readings were taken at 540 nm, and quantification was based on a standard fructose curve (0.02 to 5 mg/mL).

### 2.4. Monomeric Composition

For monosaccharide determination in the mezcal-vinasse sample, the procedure reported by Muñoz-Almagro [17] was employed. Briefly, 30 mg of sample were combined with 1.5 mL of 2 M trifluoroacetic acid (TFA) in sealed vials under a nitrogen atmosphere. The mixtures were heated at 110 °C for 4 h and subsequently subjected to vacuum evaporation. An internal standard (400 μL of 0.5 mg/mL β-phenyl glucoside) was then incorporated, followed by a second evaporation step. The resulting material was derivatized prior to GC-FID analysis, and all experiments were conducted in duplicate.

The analysis was carried out on an Agilent Technologies gas chromatograph (GC7890A) equipped with a flame ionization detector (FID) and a 7693 automatic injector. The separation of trimethylsilyl (TMSO) derivatives was performed on a DB-5HT fused silica capillary column (30 m × 0.32 mm × 0.10 μm) from J & W Scientific (Folsom, CA, USA). Nitrogen was used as the carrier gas at a flow rate of 1 mL/min. The detector was set to a temperature of 280 °C, while the oven temperature was programmed to ramp from 150 °C to 380 °C at a rate of 10 °C/min. Samples were injected in split mode with a 1:20 ratio. Data were processed and integrated using the Agilent ChemStation software (Washington, DE, USA).

### 2.5. Low-Molecular-Weight Carbohydrates

Low-molecular-weight carbohydrates (LMWC) were analyzed following the methodology previously described by Muñoz-Almagro et al. [17]. A total of 30 mg of freeze-dried mezcal-vinasse was mixed with 0.2 mL of water and 0.8 mL of a phenyl-β-D-glucoside solution (0.5 mg/mL in ethanol), used as the internal standard (IS). The mixture was stirred for 30 min, followed by centrifugation for 1 min. The resulting supernatant was collected, and a second extraction was subsequently performed. The combined supernatants were evaporated, derivatized, and analyzed using a gas chromatograph (Agilent Technologies 7890A, Santa Clara, CA, USA) equipped with a flame ionization detector and a DB-5HT capillary column (30 m × 0.32 mm × 0.10 μm) from J & W Scientific (Folsom, CA, USA). Data analysis was carried out using Agilent ChemStation software (version Rev. B.03.01, Wilmington, DE, USA). Quantification was performed using standard solutions of known carbohydrates, with phenyl-β-D-glucoside as the IS.

### 2.6. Molecular Mass

The molecular mass (Mw) estimation followed Ferreira-Lazarte’s [18] protocol, involving the preparation of a sample solution of 5 mg/mL in water, subsequent dilution to 1 mg/mL and filtration through a 0.45 μm pore size filter. The filtered sample was placed in an HPSEC-ELSD vial. Analysis utilized an Agilent Technologies LC 1260 Infinity system with TSK-GEL Columns, a TSK-Gel precolumn, a mobile phase of 0.1 M ammonium acetate, a flow rate of 0.5 mL/min at 30 °C, and a 20 μL sample injection. An evaporative light scattering detector (ELSD) (Agilent Technologies, Santa Clara, CA, USA) at 30 °C and external calibration with pululan patterns were employed for Mw determination. The calibration curve had the elution volume on the *x*-axis and the logarithm of Mw on the *y*-axis, using known Mw values (805, 200, 10, 3, and 0.3 kDa) of commercial pululan patterns (Fluka Analytical) at a concentration of 1 mg/mL.

### 2.7. Free Phenolic Compounds

The extraction of free phenolic compounds was done using the following methodology. Briefly, 100 µL of vinasse was measured, to which 1 mL of methanol was added. The mixture was stirred and sonicated at 40 kHz for 30 min using a 5510 (Branson, Danbury, CT, USA). The vinasse mixture was centrifugated at 13,000× *g* (RCF)for 5 min at 25 °C with a mini spin plus (Eppendorf, Hamburg, Germany). The supernatant was collected and stored at −80 °C waiting for further use.

### 2.8. Phenolic Acids, Flavonoids and Condensed Tannins

For the determination of total phenolic compounds, the methodology proposed by [19] using the Folin–Ciocalteu reagent was followed. Results were compared against a standard curve of gallic acid with a concentration range that went from 0 to 0.01 mg/mL; the results were expressed as mg of gallic acid/g.

The quantification of flavonoids was carried out using 2-aminoethyl diphenylborinate as described by Oomah et al. [20]. Results were compared against a standard curve of rutin with a concentration range that went from 0 to 50 µg/mL. Results were expressed as mg of rutin/g.

Condensed tannins were quantified using a vanillin–hydrochloric acid solution according to the method outlined by Deshpande and Cheryan [21]. Results were compared against a catechin standard curve with a concentration range that went from 0.1 to 0.8 mg/mL.

### 2.9. Phenolic Profile

To characterize the phenolic composition of mezcal-vinasse, samples were initially passed through a 0.45 µm membrane to eliminate suspended particles. Chromatographic separation was performed using an Agilent Technologies 1100 Series HPLC system (Palo Alto, CA, USA) equipped with a diode array detector (DAD) for multi-wavelength acquisition. Separation of compounds was carried out on a Zorbax Eclipse XDB-C18 column (4.6 × 250 mm, 5 µm particle size) operating under reversed-phase conditions. The mobile phase consisted of water containing 1% acetic acid (*v*/*v*) and acetonitrile with 1% acetic acid (*v*/*v*), using a gradient elution program based on Ramírez-Jiménez et al. [22] with minor adjustments. The flow rate was set at 1.0 mL/min, and analyses were conducted at ambient temperature. Detection wavelengths were fixed at 280 nm for phenolic acids and 320 nm for flavonoids, while full spectra (190–600 nm) were simultaneously recorded to support compound identification. Quantification was achieved through calibration curves ranging from 0.05 to 0.1 mg/mL, prepared using standards such as gallic acid, catechin, ferulic acid, ellagic acid, rutin, and kaempferol. The calibration curves exhibited strong linearity, with coefficients of determination (R^2^) between 0.95 and 0.99. Compound identification was performed by comparing retention times and UV–Vis spectra against those of the corresponding standards.

### 2.10. Volatile Compounds

Volatile compounds were isolated using a liquid–liquid extraction procedure based on Prado-Jaramillo et al. [23], employing dichloromethane as the extraction solvent. In brief, vinasse samples were transferred into 40 mL centrifuge tubes, followed by the addition of 10 mL of CH_2_Cl_2_. The mixtures were agitated for 5 min and then centrifuged at 5000 rpm for 10 min at 10 °C. After phase separation, the organic fraction was collected and dried using anhydrous Na_2_SO_4_. The extracts were subsequently concentrated to a final volume of 1.5 mL with a rotary evaporator. The concentrated solutions were placed in amber vials and stored at −20 °C until chromatographic evaluation. All vinasse samples were extracted and analyzed in triplicate.

The separation and identification of volatile compounds were carried out using a gas chromatograph coupled with a selective mass spectrometer detector. Phenyl-β-D-glucoside was used as an internal standard for quantification. An HP-FFAP capillary column (25 m × 0.32 mm i.d., 0.52 µm film thickness; Agilent Technologies) was employed for the separation. Helium was used as the carrier gas at a constant flow of 2 mL/min. The oven program was set as follows: an initial temperature of 40 °C for 5 min, then ramped at 20 °C/min to 100 °C (held for 1 min), followed by 3 °C/min to 230 °C (held for 40 min). The injector temperature was 220 °C, and injections were performed in splitless mode. The mass spectrometer operated with electron impact ionization at 70 eV and 260 °C. Compound identification was based on comparison of mass spectra with the NIST library.

### 2.11. Antioxidant Capacity

The antioxidant capacity was evaluated using two radical scavenging assays: 2,2′-azino-bis(3-ethylbenzothiazoline-6-sulfonic acid) (ABTS) as described by Nenadis et al. [24], and 2,2-diphenyl-1-picrylhydrazyl (DPPH) following the method of Fukumoto and Mazza [25]. The results were quantified using a Trolox standard curve ranging from 20 to 800 mM as a reference.

### 2.12. Process-Generated and Antinutritional Compounds

#### 2.12.1. Furfural and HMF

Furfural and HMF concentrations in mezcal-vinasse were quantified using high-performance liquid chromatography (HPLC), using a modified protocol based on Makawi et al. [26] method, including an aqueous extraction for the furfural and HMF fractions. The analyses were conducted with an HPLC system equipped with a diode array detector (DAD) and operated under isocratic conditions. Separation was achieved using a Zorbax Eclipse XDB-C18 column (250 mm × 4.6 mm, 5 µm; Agilent Technologies), which was maintained at a temperature of 30 °C. The mobile phase consisted of acetonitrile and water in an 80:20 (*v*/*v*) ratio, delivered at a 1.0 mL/min flow rate. Each sample had an injection volume of 20 µL. Detection was carried out with the DAD set to 277 nm for furfural and 285 nm for HMF. The total run time for each analysis was 15 min. Quantification was based on external standard calibration curves ranging from 1.0 to 30.0 mmol of HMF and 1.0 to 12.0 mmol of furfural. Calibration curves showed linearity, with coefficients of determination (R^2^) greater than 0.99. Sample was analyzed in triplicate to ensure accuracy and reproducibility.

#### 2.12.2. Condensed Tannins

Condensed tannins in mezcal-vinasse were quantified using the method described by Deshpande and Cheryan [21]. Reagents included 8% HCl (21.6 mL in 100 mL methanol), 4% HCl (10.8 mL in 100 mL methanol), 1% vanillin (0.5 g in 50 mL methanol, protected from light), and a freshly prepared (+)-catechin stock solution (0.8 mg/mL in methanol). A standard curve was generated using catechin concentrations ranging from 0.02 to 0.8 mg/mL. For the assay, 50 µL of each standard or methanolic vinasse extract also used for the phenolic quantification was combined with 200 µL of a 1:1 vanillin–HCl (8%) solution in a microplate. A blank was prepared with 4% HCl and methanol. Samples were incubated for 10 min in the dark, and the absorbance was recorded at 492 nm.

#### 2.12.3. Oxalates

Oxalate content in mezcal-vinasse was determined using the titrimetric method by Day and Underwood [27]. A 1 g sample was mixed with 50 mL of 1.5 M sulfuric acid and agitated for 1 h. The mixture was then filtered using Whatman No. 1 paper and centrifuged at 10,000 rpm for 10 min. A 25 mL aliquot of the supernatant was recovered and titrated with 0.05 M potassium permanganate (KMnO_4_) at 80 °C until a stable pink color persisted for 30 s. Results are expressed as equivalents of sodium oxalate per gram of liquid sample.

### 2.13. Microbiological Analysis

Microbiological analyses were performed on vinasse, which covered *Salmonella* spp., total coliforms, aerobic mesophiles, fungi and yeasts according to the methodology described in the NOM-210-SSA1-2014, NOM-092-SSA1-1994 and NOM-111-SSA1-1994 [28,29,30]. The microbiological analyses were carried out with the collaboration of the Laboratory for the Evaluation and Control of Microbial Risks in Food of the Autonomous University of Querétaro.

### 2.14. Cytotoxicity Analysis

Cytotoxicity analysis was performed with the CellTiter 96^®^ Aqueous One Solution Cell Proliferation Assay Kit (Promega, Madison, WI, United States). Briefly, cells of the human epithelial colorectal adenocarcinoma Caco2 cell line were grown in DMEM culture medium with 10% fetal bovine serum added until a confluence of 85% was reached. The cells were detached and placed in a 96-well box at a concentration of 5000 cells per well and allowed to adhere for 24 h. Subsequently, phenolic extracts obtained from mezcal vinasse were added at concentrations of 100 mg/mL up to 20 mg/mL and interaction was allowed for 24 h. At the end of the time, 20 µL of CellTiter solution was added to each well, incubated for 1 h, and the absorbance was read at a wavelength of 570 nm(BIO-RAD, xMark Microplate Spectophotometer) (Bio-Rad Laboratories, Hercules, CA, USA). Each treatment was performed in triplicate. The absorbance was directly proportional to the number of living cells, and viability was expressed as a percentage relative to control (untreated cells). The cell line Caco-2 was kindly provided by the Cell Culture Laboratory at Tecnológico de Monterrey, Monterrey Campus (Monterrey, Mexico). This cell line originally derived from ATCC (HTB-37). Cells were maintained under standard culture conditions and used for the experiments described above.

### 2.15. Statistical Analysis

All measurements were conducted in triplicate, and the results are reported as mean values. Statistical analysis included one-way ANOVA, using a significance threshold of 0.05. Differences between means were evaluated using Tukey’s post hoc test. Prior to these analyses, data sets were tested for normality and homogeneity. All statistical procedures were performed using Minitab Statistical Software (Minitab Version 21, LLC., 2021, State College, PA, USA). Differences between means were considered statistically significant at *p* < 0.05.

## 3. Results and Discussion

### 3.1. Proximate Characterization

Mezcal vinasse exhibited an acidic pH of 3.48 ± 0.03, which is characteristic of distillation residues and has important implications for microbial stability, storage, and downstream treatment strategies [7]. The proximate composition of mezcal-vinasse (Table 1) is a critical parameter for evaluating the potential for use and valorization of this waste of the mezcal industry. A high water content, representing 96%, was observed in agave vinasse sample. This high moisture content is typical of liquid wastes from fermentation and distillation processes and can affect the vinasse’s stability and potential for treatment or valorization [31]. A high moisture content favors microbial activity and promotes putrefaction [3].

The ash fraction (10.69% in the dry basis) reflects a high concentration of minerals and organic salts. Rodriguez-Felix et al. [32] reported that the most prevalent minerals observed in tequila vinasse were potassium, calcium, and magnesium, which can be attributed to the agave substrate. The protein content reported was 4.86% in dry basis, with a limited nitrogen contribution as compared to other agro-industrial waste. This content could be attributed to lysed microorganisms from the fermentation process. Another source of protein could be a limited generation of single-cell protein produced during the fermentation process [33]. However, precise data on the proximate composition of mezcal-vinasse, including protein, lipids, and carbohydrates, remain scarce in the literature. Other vinasses, such as sugarcane vinasse, have a protein content of around 9.1% in dry basis; however, these values may be overestimated, as nitrogen is mainly found in the form of non-protein nitrogen, such as ammonia and free amino acids, and is poorly utilized by monogastric animals [34]; this may also be the case for mezcal vinasses.

The lipid content is low (0.24%), since during the typical fermentation of by-products, most of the energy substrates are metabolized into alcohols and volatiles rather than being stored as lipids [3]. Finally, the carbohydrate fraction presents the highest value among the dry fraction of the sample (84.22% in dry basis). This may be due to the presence of residual sugars or sugar-derived compounds, such as oligosaccharides and breakdown products from agave cell walls, and may also be related to the soluble and insoluble fiber content [31,35]. This composition of mezcal-vinasse (low protein and fat content vs. high levels of minerals and residual sugars) makes it interesting to explore other areas for their potential use and revalorization.

Results are presented as mean ± standard deviation, and experiments were performed in triplicate.

### 3.2. Carbohydrate Profile

**Figure 2:**
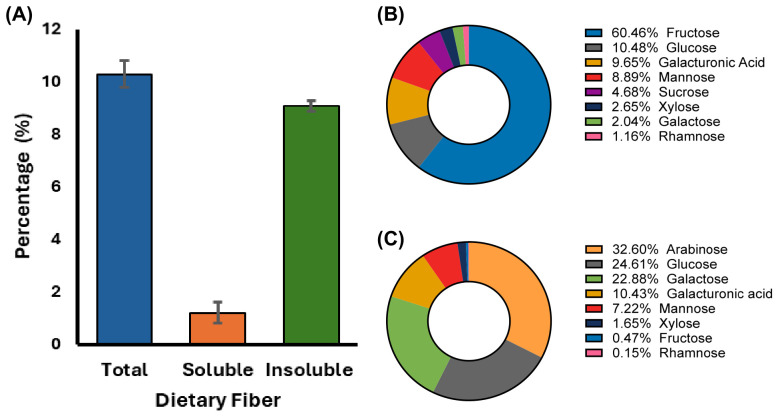
Carbohydrate composition of mezcal vinasse. (**A**) Fiber profile. (**B**) Low-molecular-weight carbohydrates. (**C**) Monomeric carbohydrate composition. Values are expressed on a dry basis. Results are presented as mean ± standard deviation for (**A**) and as mean values for (**B**,**C**). All experiments were performed in triplicate.

The fiber composition of mezcal-vinasse is presented in Figure 2A. The total dietary fiber (TDF) content was 10.3% of the carbohydrate fraction, where the soluble fiber (SF) content was 1.2%, and the insoluble fiber (IF) fraction accounted for most of the fiber content, with a value of 9.1%. These results shows that the fiber present in mezcal-vinasse is predominantly insoluble, which may influence its potential applications in food formulations, particularly in improving intestinal transit and water retention capacity [36]. When comparing the soluble to insoluble fiber ratio of mezcal-vinasse, it exhibits a ratio of approximately 1:8, notably higher in insoluble fiber compared to many common foods. For instance, wheat bran typically has a soluble to insoluble fiber ratio of about 1:3, and oats have a ratio close to 1:1.5. [37]. The use of both types of fiber, soluble and insoluble, in food formulations is a common strategy to combine the characteristic effects of each type of fiber, potentially improving the texture and nutritional profile of high-fiber foods [38].

### 3.3. Low-Molecular-Weight Carbohydrates

Low-molecular-weight carbohydrates (LMWCs) include monosaccharides, disaccharides, and oligosaccharides that are obtained by aqueous extraction with agitation. These compounds are located in cytoplasmic and vacuolar compartments, in extracellular spaces or weakly attached to the cell wall, and participate in processes of osmoregulation, energy reserve, and cell signaling, as well as acting as precursors of secondary metabolism [39]. No previous studies have investigated the composition of low-molecular-weight carbohydrates aiming to use them in the food sector as a potential functional ingredient. In this study (Figure 2B), the following concentration order was observed: fructose > glucose > galacturonic acid > mannose > sucrose > xylose > galactose > rhamnose.

Mezcal vinasse is primarily composed of water, non-volatile compounds, phenolic compounds, high concentrations of minerals, organic acids, ethanol, light alcohols, and unfermented residual sugars [7]. The findings reveal that the predominant low-molecular-weight carbohydrates in mezcal vinasse are residual sugars—unfermented monosaccharides and disaccharides that persist due to suboptimal fermentation conditions, including microbial limitations, pH imbalance, temperature fluctuations, and the presence of inhibitory compounds [40].

In a study conducted on several samples of juice of *Agave* species, including *A. americana*, *A. tequilana*, and *A. salmiana*, glucose was identified as the predominant sugar monomer, followed by fructose and galactose [41]. Similarly, in juice extracted from steam *A. americana*, glucose was reported as the most abundant monomer, followed by fructose [42]. Conversely, in a more recent study on *Agave* sp. juice, fructose was found to be the most concentrated sugar, followed by glucose [43]. These discrepancies in sugar composition may be attributed to several factors, including agave species, environmental conditions, plant maturity, and the specific processing methods employed in mezcal production [2].

The low-molecular-weight carbohydrates present in mezcal hold significant potential for valorization in the food industry given the fact that free sugars found in the medium are easy to recover, after which they can be used as natural sweeteners, or substrates for fermentation processes for producing value-added food ingredients [44]. The presence of high concentrations of residual sugars may be associated with incomplete fermentation, potentially influenced by process variability, microbial activity, or inhibitory conditions such as low pH and the presence of fermentation by-products.

### 3.4. Monomeric Profile

The monomeric composition of carbohydrates of mezcal were obtained after acid hydrolysis, leaving exclusively individual monosaccharides. The monomeric composition of mezcal-vinasse derived from mezcal production has not been previously reported. Accordingly, this study presents (Figure 2C) the first characterization of its monomeric profile, which was found to be: arabinose > glucose > galactose > galacturonic acid > mannose > xylose > fructose > rhamnose. This monosaccharide profile can be used to infer which plant cell wall polysaccharides are present in the vinasse. The monosaccharide pattern points to pectins as the dominant polysaccharide fraction, since the high proportions of arabinose and galactose are indicative of an abundance of arabinans and galactans, characteristic components of the rhamnogalacturonan I (RG-I) side chains, while the high galacturonic acid content suggests the presence of homogalacturonan (HG) [45]. Meanwhile, the low proportion of rhamnose indicates that the RG-I backbone is present but in a lower relative proportion compared to HG and the neutral sugar-rich side chains [46]. In the analyzed vinasse samples, the presence of a fraction of hemicelluloses and pectins can be predicted, including arabinoxylans, glucomannans, and galactomannans, as indicated by the abundance of arabinose, xylose, glucose, mannose and galactose. A smaller contribution of cellulose is also evident, manifested by free or partially hydrolyzed glucose [47].

Understanding the monomeric composition of mezcal-vinasse provides relevant insights for its biotechnological and food-oriented valorization, as these structural features influence fermentability, bioaccessibility, and interactions with microbial systems. This information is essential for evaluating its potential use as a substrate for microbial processes, the development of functional ingredients, and the design of appropriate stabilization or processing strategies. Furthermore, having this information is crucial for implementing proper treatment and open the doors to their studies for potential revalorization and subsequent re-inclusion for human consumption. Notably, residual sugars can act as precursors for furanic compound formation under thermal conditions, linking carbohydrate composition with the generation of HMF and furfural observed in vinasse

### 3.5. Bioactive Compounds

**Figure 3:**
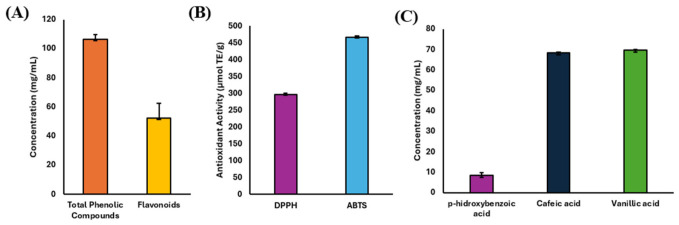
(**A**) Flavonoid content expressed as gallic acid equivalents and phenolic content expressed as rutin equivalents in mezcal-vinasse. (**B**) Antioxidant activity of mezcal-vinasse. (**C**) Phenolic compounds found in mezcal-vinasse. Results are presented as mean ± standard deviation, and experiments were performed in triplicate.

The total phenolic and flavonoid contents, as well as the antioxidant activity of mezcal-vinasse, are presented in Figure 3. The total phenolic content (TPC) was 106.5 ± 0.03 mg/L. The flavonoid content was found to be 52.47 ± 9.80 mg/L, suggesting that about half of the proportion of the phenolic compounds in vinasse are flavonoids, which are known for their health-promoting effects, such as antioxidant and anti-inflammatory capabilities [2]. These results contrast with those reported by Bailón-Pérez [48], who reported a phenol concentration of tequila vinasse of 1.30 ± 0.08 mg/L, which is lower than the concentration observed in our study; tequila vinasse is used here as a reference matrix due to the limited availability of compositional data for mezcal vinasse. Notably this variability was also observed by Rodriguez-Félix [32], who reported a range of concentrations in tequila vinasse ranging from 4 to 100 mg/L. These discrepancies may uncover the influence of different external factors, such as agave species, production processes, and analytical methods, on the phenolic composition.

The phenolic composition of mezcal contributes to the sensory characteristics and potential health benefits of the beverages. In mezcal, phenolic compounds such as 3-ethyl-phenol have been identified, contributing to its characteristic smoky flavor [49]. Due to the limited availability of compositional data for mezcal vinasse, comparisons are sometimes made with related matrices; however, the differences between beverages and vinasse should be considered; furthermore, tequila contains various phenolic acids, including gallic, vanillic, and syringic acids. Notably, the TPC in white tequilas, as reported by Alcazár Magaña [50], ranges from 36 to 408 μg/L, while rested and aged tequilas exhibit higher concentrations, ranging from 515 to 4296 μg/L and 2048 to 3249 μg/L, respectively. These variations could be attributed to the extraction of phenolic compounds from oak barrels during the aging process, which enhances the complexity and depth of the flavor profile [51]. Notably, the variation in TPC in mezcal has also been attributed to aging and storing. Ávila Reyes [51] detected a total phenolic compounds content in mezcal ranging from 179 to 938 mg/L, increasing with the aging time.

The presence of p-hydroxybenzoic acid, caffeic acid, and vanillic acid in the mezcal vinasse confirms the release of low-molecular-weight phenolic compounds that may be associated with the partial degradation of lignin and other structural components of the agave during fermentation [52]. These compounds can be grouped into two families: benzoic acid derivatives (p-hydroxybenzoic and vanillic acid) and cinnamic acid derivatives (caffeic), which share the ability to donate electrons thanks to their aromatic hydroxyl groups, contributing to antioxidant activity [53,54]. Caffeic acid, with an additional conjugated system, tends to show greater antioxidant capacity and biological activity, including anti-inflammatory and antimicrobial properties [55]. Vanillic acid is associated with the degradation of more complex compounds and has been linked to potential anticancer activity, while p-hydroxybenzoic acid is a typical marker of lignin degradation processes in plant matrices [52]. Overall, the detection of these phenols reinforces the potential of vinasse as a source of bioactive compounds with a functional effect.

The antioxidant activity was evaluated using DPPH and ABTS assays. The DPPH radical scavenging activity was 296.36 ± 0.02 µmol TE/g, while the ABTS radical scavenging activity was 465.5 ± 0.04 µmol TE/g. These results are in agreement with the findings of Molina-Cortes [56], where the antioxidant capacities of sugarcane vinasse were measured, obtaining a concentration of 175.65 µmol/g and 377.1 µmol/g in the scavenger tests of DPPH and ABTS respectively. The slightly higher antioxidant activity observed in this study could be attributed to differences in raw materials, processing conditions, or inherent compositional variations between mezcal and sugarcane vinasse. The higher ABTS value compared to DPPH suggests that the phenolic compounds in vinasse may have a greater capacity to scavenge hydrophilic radicals [57]. The difference between the DPPH and ABTS assays can be explained by the solubility and reactivity of phenolic compounds towards the radicals used in each test. ABTS is considered more suitable for evaluating hydrophilic antioxidants, while DPPH preferentially interacts with lipophilic compounds [58]. Because of this, the higher ABTS scavenging activity suggests that the antioxidant compounds in vinasse are predominantly hydrophilic in nature. The phenolic content may be related to the breakdown of lignocellulosic structures in agave during thermal processing, leading to the release of bound phenolic compounds into the vinasse matrix. Collectively, the high phenolic content and antioxidant activity of mezcal vinasse support its revalorization as a functional and biotechnological resource.

### 3.6. Volatile Compounds GC

The volatile profile of mezcal vinasse is presented in Table 2, revealing a mix of fermentation-derived metabolites and degradation by-products with both functional and safety implications. Acetoin, a microbial signaling compound, promotes plant growth and stress resilience [59]. Its counterpart, 2,3-butanediol, is recognized as a valuable platform chemical obtained via microbial fermentation, with applications ranging from biofuels to cosmetics [60]. Acetic acid, the most prevalent volatile compound found, has an antimicrobial agent role and function as a modulator of gut and inflammatory responses [61]. Aromatic volatiles such as phenylethyl alcohol show clear antimicrobial activity, affecting bacterial membranes [62]. The presence of HMF points to thermal sugar degradation and raises health concerns, as this is a known heat-born food toxicant [63]. Together, these findings highlight the dual nature of vinasse volatiles, encompassing compounds with beneficial functional potential and others requiring monitoring to mitigate toxicity risks. Comparative data on the volatile composition of vinasse, particularly from mezcal systems, remain scarce in the literature, limiting direct comparisons with similar matrices. As a result, the discussion focuses on the functional relevance of the identified compounds rather than direct comparison with other vinasse systems

### 3.7. Antinutritional and Process-Generated Compounds

The antinutritional and process-generated compounds obtained in mezcal-vinasse were studied, and the results are presented in Table 3. Among the compounds analyzed, oxalates were present at a concentration of 14.6 ± 1.60 mg sodium oxalate/g dry sample. Oxalates are known to interfere with mineral absorption and can form insoluble complexes, potentially impacting soil and water quality if vinasse is improperly managed [66], their presence in the vinasse matrix may be an indication of the need for caution in potential applications involving land disposal or agricultural reuse.

Tannins were identified as the most abundant antinutritional factor, with a concentration of 15.3 ± 0.80 mg catechin/g dry sample. While tannins can have some beneficial properties at low concentrations, such as antioxidant activity, their higher levels can limit protein digestibility and reduce nutrient availability, posing challenges for both environmental disposal and potential feed applications [67]. It is recognized that a daily intake of tannin below the range of 1500–2500 mg is safe for consumption [68]; in this sense, the concentration of tannins found in mezcal vinasse should not affect human health.

Furanic compounds, specifically HMF and furfural, were also detected. HMF was found at 3830.0 ± 11.2 mg/L, while furfural was present at a lower concentration of 160.0 ± 1.7 mg/L. These values are high when compared to previous studies; for instance, Rodríguez-Félix et al. [32] reported 52.1 mg/L of furfural and 347.6 mg/L of HMF in tequila vinasse, and Lorenzo-Santiago et al. [69] observed increases in these compounds following thermal treatments. The elevated levels in mezcal vinasse are likely due to thermal treatment during agave cooking, leading to the Maillard reaction; notably, the monomeric composition of mezcal vinasse provides important insights into the formation of furanic compounds. Hexose sugars such as glucose and fructose are known precursors of 5-hydroxymethylfurfural (HMF) through dehydration reactions under acidic and thermal conditions, while pentose sugars such as arabinose and xylose can lead to the formation of furfural [70]. Given the predominance of arabinose and glucose in the analyzed samples, the observed concentrations of both HMF and furfural can be directly linked to the degradation of these monosaccharides during the agave cooking stage, where high temperatures and acidic conditions promote these transformations. The concentration of these compounds should be controlled; thus, in Mexico, NOM-070-SCFI-2016 [71] establishes a maximum limit of 5 mg/100 g of alcohol for furfural in mezcal. HMF has been attributed to cause a loss in digestibility for proteins and inhibition of enzymes, along with bioavailability interference for some minerals at high concentrations (100 mg/kg of body weight) [72,73]; although regulatory limits for compounds such as furfural are established for mezcal rather than vinasse, these thresholds provide a useful reference framework when considering the potential revalorization of vinasse for food-related applications. Moreover, diets enriched in Maillard reaction products containing approximately 3.9 mg HMF·kg^−1^ resulted in a ~6% decrease in apparent protein digestibility and a ~12% reduction in nitrogen absorption compared with low-HMF diets (~0.9 mg HMF·kg^−1^) in human crossover studies [74]. In parallel, Maillard-rich diets significantly reduced iron bioavailability (~2.7-fold) and increased fecal iron excretion [75]. These findings underscore the complexity of vinasse valorization: as the presence of furanic compounds, along with tannins and oxalates, necessitates careful consideration for reuse strategies to mitigate environmental risks, the reduction in this furanic compounds may be an interesting and desirable approach to continue the path towards the revalorization of mezcal vinasses. Although specific regulatory limits for HMF and furfural in vinasse are not established, toxicological studies indicate that high concentrations of these compounds may pose risks to human health. Therefore, their levels in vinasse highlight the need for mitigation strategies prior to any potential food-related application.

Results are presented as mean ± standard deviation, and experiments were performed in triplicate.

### 3.8. Microbiological Analysis

The microbiological analysis of mezcal-vinasse samples is presented in Table 4. The results indicate a bacterial mesophilic aerobic (BMA) count of 120 CFU/mL, suggesting the presence of a moderate microbial load. However, mesophilic bacteria, yeasts, and total coliforms were all detected at levels below the quantification limit (<10 CFU/mL), indicating minimal contamination by these microorganisms. Notably, *Salmonella* was absent from the sample. These findings are consistent with previous studies on tequila vinasse, which have reported acidic pH values ranging from 3.9 to 5.1 and high COD levels between 50,000 and 95,000 mg/L, conditions that can limit microbial proliferation [76]. The low microbial concentration observed suggests that the physicochemical properties of mezcal-vinasse may inhibit the growth of microorganisms. This may be a consideration for their potential biotechnological applications.

### 3.9. Cytotoxicity Assay

**Figure 4:**
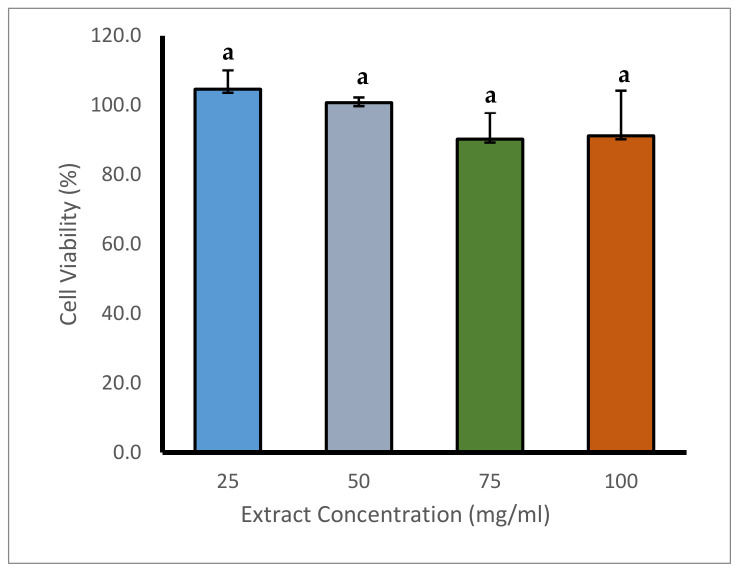
Cytotoxicity assay for phenolic extraction of mezcal-vinasse. Means sharing the same letter indicate no significant difference, Statistical significance was evaluated at *p* < 0.05.

The results shown in Figure 4 show that the phenolic fraction extracted from Mezcal-vinasse had no significant differences (*p* > 0.05), as determined by one-way ANOVA, between treated and control cells on CaCo-2 cell line, even with high concentrations (25–100 mg/mL), maintaining a cellular viability above 90%. This finding suggests that these phenolic compounds fraction could be safe in an in vitro model, warranting further validation in biological models for nutraceuticals and pharmaceutical applications, especially in the gut health context.

This behavior is consistent with previous studies that evaluated phenolic extracts from vegetal sources. Mahmood et al. [77] report that phenolic and terpenic fractions of *Prunus arabica* show antiproliferative effects in cancer cell lines without inducing significant cytotoxicity in regular human fibroblasts. Additionally, studies on *A. lechuguilla* extracts showed moderate cytotoxicity in the HeLa cell line (IC_50_ between 78 y 95 µg/mL), but low impact in regular healthy cell lines such as Vero [78]. In comparison, our results demonstrate that even at higher concentrations, the phenolic fraction extracted from agave vinasse does not affect negatively the cell viability of CaCo2 cell line, suggesting a phenolic fraction with low reactivity towards non-malignant cells.

Furthermore, phenolic extracts obtained from by-products such as grape pomace have shown antiproliferative activity in CaCo2 cells, with viability reductions of up to 27% at concentrations of 75 µg/mL [79]. In contrast, the phenolic fraction evaluated in our study did not present such an effect, which could be attributed to differences in the chemical composition of the extracts, such as the type and degree of polymerization of the phenolic compounds, as well as the presence of other metabolites with protective or modulatory activity. Some phenolic compounds may contribute to intestinal barrier integrity by modulating tight junction proteins (claudins, occludins, ZO-1) in intestinal epithelial cells, while also enhancing antioxidant defenses via activation of the Nrf2 pathway and upregulation of enzymes such as HO-1 and glutathione peroxidase [80,81]

In this context, it is relevant to consider the presence of furfural compounds, such as HMF, in the analyzed phenolic fraction. Studies have shown that, at high concentrations, furfurals can induce cytotoxic effects through mechanisms related to oxidative stress, loss of mitochondrial potential, and DNA fragmentation in cell lines such as HepG2 and Caco2. However, at low doses, they may exhibit hormetic activity that stimulates cellular antioxidant pathways [82]. In addition, it has been reported that some phenols can negatively modulate HMF toxicity by activating the Nrf2/ARE pathway, promoting the expression of detoxifying genes such as NQO1 and HO-1 [82]. Nevertheless, it is essential to highlight the need to reduce these furanic compounds to concentrations below the limits established by the NOM in order to enable the valorization of mezcal vinasse and its potential reintegration into the food chain or for general consumption.

## 4. Conclusions

This study provides a comprehensive physicochemical, compositional and bioactive characterization of mezcal-vinasse derived from *Agave angustifolia*, including detailed carbohydrate profiling, phenolic and flavonoid quantification, antioxidant activity, and the identification of antinutritional and process-generated compounds. The results reveal a matrix rich in carbohydrates and phenolic compounds, with significant antioxidant activity. These observations allow us to start investigating the use of mezcal-vinasse as a potential source of bioactive compounds, encouraging further study aiming to achieve its revalorization. This study allowed for a reduction in the gap of knowledge regarding the use of agave vinasse as a potential ingredient with future applications in the food sector.

However, the quantification of antinutritional and process-generated compounds such as tannins, oxalates, and furanic derivatives underscores the importance of careful management strategies for both environmental safety and human health. The elevated concentrations of HMF and furfural, likely originating from thermal processing during mezcal production, may pose constraints on its direct reuse without prior treatment.

This study is limited to a single vinasse source, and variability associated with agave species and production conditions was not evaluated. Therefore, future research should focus on the assessment of compositional variability across different production systems, the development of detoxification strategies, and the validation of the functional properties of vinasse in biological models. In that regard, these findings lay a foundation for the development of targeted mitigation and processing strategies that could enable the safe and sustainable use of mezcal-vinasse as a functional ingredient in the future.

## Figures and Tables

**Table 1:** Proximate characterization, antinutritional and process-generated compounds, and microbiological analysis of mezcal-vinasse. All compositional values are expressed on a dry basis.

Proximate Composition	
Ashes (%)	10.69 ± 0.28
Protein (%)	4.86 ± 0.34
Lipids (%)	0.24 ± 0.04
Carbohydrates (%)	84.22 ± 0.18
pH	3.48 ± 0.03

**Table 2:** Overview of volatile compounds identified by gas chromatography in mezcal-vinasse and their reported bioactivities.

Compound	Retention Time	Relative Abundance (%)	MW	Ion Fragments	Type	Bioactivity	Cite
2-Butanone, 3-hydroxy (Acetoin)	15.19	9.86	88.104	45.999, 43.556, 88.990	Ketone alcohol	Microbial signal, antioxidant	[59]
Acetic acid	29.88	31.63	60.052	43.999, 45.903, 60.747	Short-chain fatty acid	Antimicrobial, gut health, anti-inflammatory	[61]
2,3-Butanediol	37.38	5.3	90.121	45.999, 43.129, 29.102	Diol	Moisturizer, fermentation by product	[60]
Phenylethyl Alcohol	52.74	26.8	122.160	91.999, 92.557, 65.228	Aromatic alcohol	Antimicrobial, neuroactive,	[62]
Phenol	56.09	0.9	94.110	94.999, 66.387, 65.266	Aromatic compound	Antioxidant at low dose,	[64]
						toxic at high dose	
2-Methyl-trans-hexadienedioic acid	61.32	5.8	156.160	125.999, 97.900, 111.490	Dicarboxylic acid	Antioxidant or lipid-degradation indicator	[65]
5-Hydroxymethyl furfural	71.89	8.95	126.110	97.999, 126.779, 41.732	Furfural derivative	Cytotoxic at high levels	[63]

**Table 3:** Antinutritional and process-derived compounds of mezcal-vinasse.

Antinutritional and Undesired Process-Derived Compounds	
Tannins (mg catequin/g)	15.3 ± 0.80
Oxalate (mg sodium oxalate/g)	14.6 ± 1.60
Hydroxymethyl furfural (mg/L)	3830.0 ± 11.2
Furfural (mg/L)	160.3 ± 1.7

**Table 4:** Microbiological analysis of mezcal-vinasse.

Microbiological Analysis	
BMA (CFU/mL)	120
Mesophiles (CFU/mL)	<10
Yeasts (CFU/mL)	<10
Total Coliforms (CFU/mL)	<10
*Salmonella*	Absent

## Data Availability

The original contributions presented in this study are included in the article. Further inquiries can be directed to the corresponding author.

## Abbreviations

The following abbreviations are used in this manuscript:

SAGARPA	Secretaría de Agricultura, Ganadería, Desarrollo Rural, Pesca y Alimentación
PDO	Protected Designation of Origin
CETESB	Companhia Ambiental do Estado de São Paulo
COD	Chemical oxygen demand
BOD	Biological oxygen demand
HMF	5-hydroxy methylfurfural
S.E.	Ministry of economy
Mw	Molecular mass
TDF	Total dietary fiber
SF	Soluble fiber
IF	Insoluble fiber
LMWCs	Low-molecular-weight carbohydrates
TPC	Total phenolic content
